# Eliminating Lymphatic Filariasis, Onchocerciasis, and Schistosomiasis from the Americas: Breaking a Historical Legacy of Slavery

**DOI:** 10.1371/journal.pntd.0000071

**Published:** 2007-11-07

**Authors:** Patrick J. Lammie, John F. Lindo, W. Evan Secor, Javier Vasquez, Steven K. Ault, Mark L. Eberhard

**Affiliations:** 1 Division of Parasitic Diseases, Centers for Disease Control and Prevention, Atlanta, Georgia, United States of America; 2 Pan American Health Organization/World Health Organization, Washington, D. C., United States of America; 3 The University of the West Indies, Kingston, Jamaica; Case Western Reserve University School of Medicine, United States of America

The 2007 bicentennial of the enactment of the bill to abolish the transatlantic slave trade in the British Empire has focused new attention on the historical legacy of slavery and its human and social consequences. Beyond the societal impact of slavery, which continues to the present day, the transportation of millions of persons from sub-Saharan Africa to the New World also resulted in the importation of a number of parasitic diseases to the Americas. Lymphatic filariasis (LF), schistosomiasis, and onchocerciasis were most likely imported to the Americas via the slave trade; the presence of competent vectors (such as mosquitoes, black flies, and snails) allowed transmission and dispersal of the parasites [1]–[4]. Although parasitologists first speculated more than a century ago that the slave trade was responsible for introduction of these diseases to the New World [3], only recently has convincing evidence supporting this hypothesis been generated. Molecular evaluation of *Schistosoma mansoni* and *Onchocerca volvulus* has demonstrated very limited heterogeneity among New World strains, which share a strong affinity with West African strains of these parasites [5],[6]. Poverty and poor living conditions continue to put people at risk of LF, schistosomiasis, and onchocerciasis. On the 200th anniversary of the abolition of the slave trade, a fitting commemoration would be a commitment to the regional eradication of these three diseases that remain as a historical legacy of slavery.

Lymphatic filariasis is a mosquito-transmitted infection that is associated with the development of debilitating lymphedema, elephantiasis, and hydrocele. The principal mosquito vector in the Americas, *Culex quinquefasciatus* , breeds in polluted water and is adapted to urban environments. LF once was widely distributed throughout the region, particularly in areas where the local geography was amenable to plantation-based agriculture and large numbers of slaves were imported. Economic development in the United States and in the Caribbean led to the spontaneous disappearance of LF in some areas and substantial declines in the prevalence of infection in others [7]. Ongoing transmission in the Western Hemisphere, now limited to four countries (Brazil, the Dominican Republic, Haiti, and Guyana), is concentrated in impoverished settings and appears to be a growing problem in urban slums. LF elimination programs based on the mass distribution of antifilarial drugs have been developed in all four countries and have resulted in declines in the prevalence of filarial infection ([8] and unpublished data). However, all four programs are resource challenged and none have achieved full coverage of at-risk populations.

Onchocerciasis is well recognized as a cause of blindness and skin disease due to the pathology elicited by death of microfilariae in the tissues. Transmission of onchocerciasis in the Americas is now limited to 13 foci in six countries (Mexico, Guatemala, Columbia, Brazil, Venezuela and Ecuador) [9]. The Onchocerciasis Elimination Program in the Americas is coordinating twice-yearly mass treatment with ivermectin in these foci. Impressive strides have been made in reducing the prevalence of onchocercal disease, and in some foci interruption of transmission has been achieved, leading to the decision to halt mass treatment in one focus in Guatemala. End-game strategies for completing the interruption of transmission in the remaining active foci must now be validated [10].

2007 also marks the centenary of the first description of *Schistosoma mansoni*, the causative agent of schistosomiasis in the Americas. Schistosomiasis was once widespread throughout the Caribbean and South America and was a major cause of hepatic fibrosis and death. Through selective and mass treatment campaigns, snail control efforts, and improved sanitation, the prevalence of schistosomiasis in the Americas has decreased and fatal infections are now uncommon. However, the “subtle morbidities” of anemia and altered growth and cognitive development in children caused by schistosomiasis are increasingly recognized as public health problems [11]. Millions of persons in Brazil are still at risk of infection, but the official status of schistosomiasis transmission in a number of other former slave colonies with previously documented transmission, such as Suriname and Saint Lucia, is not known [12]. The biological and epidemiological characteristics of schistosomiasis make it much more challenging to eliminate than lymphatic filariasis or onchocerciasis. Because adult worms can live within hosts for years and the diagnostic tests to detect active infection and confirm successful cure are currently inadequate as public health tools, it is possible that an elimination strategy based exclusively on drug treatment will not be successful. On the other hand, provision of safe water and sanitary improvements in the remaining endemic foci is a valid public health goal that will yield important dividends beyond schistosomiasis control. Given the substantial decline in infection prevalence that already has occurred in the region, development of the tools and strategies that will complete the task of elimination in this hemisphere deserves serious consideration.

A new focus on neglected tropical diseases (NTDs), including LF, onchocerciasis, and schistosomiasis, by the World Health Organization and the global health community holds the promise of delivering significant benefits to populations that suffer from these diseases [13]. However, little attention has been focused on the Americas, in large part because of the focal distribution of these infections and the mistaken impression that the NTDs no longer represent a public health burden in the region. Recently, several authors have drawn attention to the burden of NTDs in the Americas, and have recommended potential solutions [14]. Equally important is the recognition that these diseases in the Americas, in contrast to the situation in Africa, can actually be eliminated with proper efforts [15]. Although significant progress has been made in reducing the prevalence of LF, onchocerciasis, and schistosomiasis in the Americas, these diseases continue to represent a threat to millions of persons, particularly in economically marginalized communities.

Slavery and the slave trade have been prohibited in all their forms by international human rights declarations and treaties that also protect the life, well being, personal freedom, and the physical and mental integrity of human beings [16]. In recent years, the topic of reparations for slavery has been a contentious political topic. One of the major difficulties with this concept is that neither the perpetrators nor direct victims of the slave trade are available to make or receive restitution. On the other hand, the elimination of diseases that are a consequence of this trade would represent a tangible contribution to the health and well being of people and communities who, arguably, still suffer from the residual affects of slavery. What is needed now is renewal of political will and a dedication of new financial and technical resources to a coordinated elimination effort consistent with international human rights treaties and guidelines that recognize the “right to the highest attainable standard of physical and mental health,” which includes the prevention, treatment, and control of epidemic, endemic, occupational, and other diseases [17],[18]. Control and elimination of NTDs from endemic areas is central to ensuring the promotion and protection of human rights.

We recognize that LF, onchocerciasis, and schistosomiasis are not the only diseases to have been imported to the Western Hemisphere through the slave trade. They are, however, diseases that still cause substantial morbidity 200 years after the end of the slave trade and are particularly attractive elimination targets. In advocating for the elimination of NTDs in the Western Hemisphere, we are not ignoring the greater burden of NTDs in sub-Saharan Africa. However, as noted by several recent reports from the International Task Force for Disease Eradication, eliminating these diseases in the Americas is now feasible, whereas the situation in Africa is not so promising [15],[19]. Recent advocacy efforts for NTDs have made a strong case for the public health and economic value of these interventions and the dramatic health benefits that integrated NTD programs can provide [13]. These efforts have led to significant new financial commitments by the Bill&Melinda Gates Foundation and the United Kingdom and United States governments and to new discussions with the Global Fund to Fight AIDS, Tuberculosis and Malaria about linking NTD programs to those targeting HIV, malaria, and tuberculosis [20]. Efforts to eradicate LF, onchocerciasis, and schistosomiasis in the Americas will provide critical operational experience with strategies to interrupt transmission in residual foci and low-prevalence settings. They will also be instructive in the development of tools to conduct surveillance following the cessation of mass treatment. This experience will benefit the African programs and galvanize increased support for the elimination of NTDs in Africa by demonstrating approaches that make this an achievable goal.

**Figure 1:**
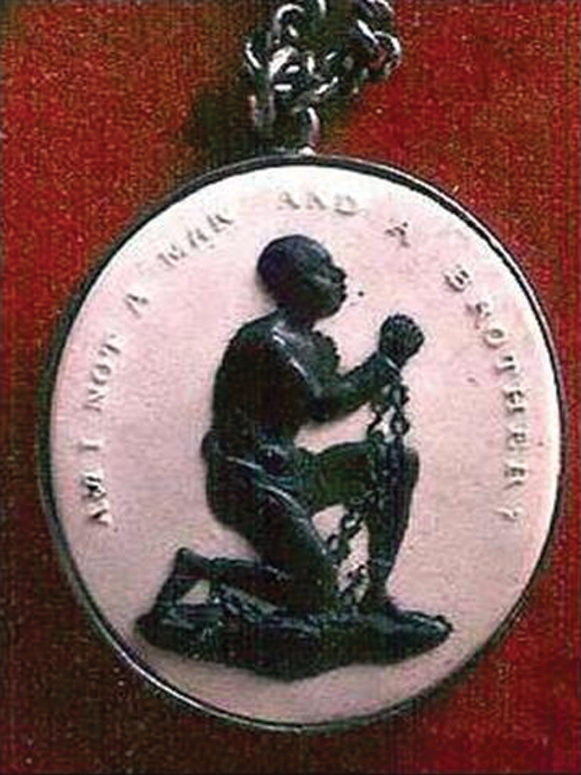
Example of a medallion designed by Josiah Wedgwood, circa 1787, to raise public awareness of the suffering brought about by the slave trade. Image used by permission of http://thepotteries.org.

Nevertheless, the global experience with guinea worm eradication portends that these efforts will not be easy. But the effort to eradicate slavery was not easy either. Slave revolts and rebellions occurred throughout the period of slavery with great loss of life on both sides. William Wilberforce worked for 20 years against great opposition to achieve passage of the law ending the British slave trade (trafficking) in 1807. And he worked another 26 years until Parliament abolished slavery itself in the British colonies. During those years, the anti-slavery proponents carried medallions similar to the ribbons and bracelets commonly worn today to signify support for various causes or diseases. On these medallions was printed “Am I not a man and a brother?” (Figure 1). Elimination of LF, onchocerciasis, and schistosomiasis in the Americas is a worthy goal for public health and for the enjoyment of physical and mental health as a basic human right, a learning tool for NTD control in more challenging settings, and a continuation of the efforts and struggles for justice of those who suffered the inhumanity of slavery and of those who worked to halt the slave trade 200 years ago. Now is the time to continue this pioneering work to eliminate three of the lasting legacies of slavery.
